# Cortical β-amyloid burden, neuropsychiatric symptoms, and cognitive status: the Mayo Clinic Study of Aging

**DOI:** 10.1038/s41398-019-0456-z

**Published:** 2019-03-28

**Authors:** Janina Krell-Roesch, Maria Vassilaki, Michelle M. Mielke, Walter K. Kremers, Val J. Lowe, Prashanthi Vemuri, Mary M. Machulda, Teresa J. Christianson, Jeremy A. Syrjanen, Gorazd B. Stokin, Lesley M. Butler, Martin Traber, Clifford R. Jack, David S. Knopman, Rosebud O. Roberts, Ronald C. Petersen, Yonas E. Geda

**Affiliations:** 1 Translational Neuroscience and Aging Laboratory, Mayo Clinic, Scottsdale, AZ USA; 2 Institute of Sports and Sports Science, Karlsruhe Institute of Technology, Karlsruhe, Germany; 3 Department of Health Sciences Research, Mayo Clinic, Rochester, MN USA; 4 Department of Neurology, Mayo Clinic, Rochester, MN USA; 5 Department of Radiology, Mayo Clinic, Rochester, MN USA; 6 Department of Psychiatry and Psychology, Mayo Clinic, Rochester, MN USA; 7 International Clinical Research Center/St. Anne Hospital, Brno, Czech Republic; 8 F. Hoffmann-La Roche Ltd, Basel, Switzerland; 9 Department of Psychiatry and Psychology, Mayo Clinic, Scottsdale, AZ USA; 10 Department of Neurology, Mayo Clinic, Scottsdale, AZ USA

## Abstract

Neuropsychiatric symptoms (NPS) are a risk factor for cognitive impairment and are associated with cortical β-amyloid (Aβ) deposition. We conducted a cross-sectional study derived from the ongoing population-based Mayo Clinic Study of Aging to examine the frequency of NPS among cognitively unimpaired (CU) and mild cognitive impairment (MCI) participants who either have normal (A−) or abnormal (A+) Aβ deposition. We also investigated whether combined presence of MCI and amyloid positivity (MCI/A+) is associated with greater odds of having NPS as compared to CU/A− (defined as reference group). Participants were 1627 CU and MCI individuals aged ≥ 50 years (54% males; median age 73 years). All participants underwent NPS assessment (Neuropsychiatric Inventory Questionnaire (NPI-Q); Beck Depression Inventory II (BDI-II); Beck Anxiety Inventory (BAI)) and ^11^C-PiB-PET. Participants with an SUVR > 1.42 were classified as A+. We conducted multivariable logistic regression analyses adjusted for age, sex, education, and APOE ε4 genotype status. The sample included 997 CU/A−, 446 CU/A+, 78 MCI/A−, and 106 MCI/A+ persons. For most NPS, the highest frequency of NPS was found in MCI/A+ and the lowest in CU/A−. The odds ratios of having NPS, depression (BDI ≥ 13), or anxiety (BAI ≥ 8, ≥ 10) were consistently highest for MCI/A+ participants. In conclusion, MCI with Aβ burden of the brain is associated with an increased risk of having NPS as compared to MCI without Aβ burden. This implies that the underlying Alzheimer’s disease biology (i.e., cerebral Aβ amyloidosis) may drive both cognitive and psychiatric symptoms.

## Introduction

Neuropsychiatric symptoms (NPS) are risk factors for mild cognitive impairment (MCI) and Alzheimer’s disease (AD) dementia^1–5^. Furthermore, there is growing evidence of an association between NPS and neuroimaging biomarker abnormality in the context of brain aging.

Although some studies have examined the associations between NPS, particularly depression and anxiety, and cortical amyloid deposition among cognitively normal^6–8^ or impaired participants^9–14^, these studies have been limited by small sample sizes, have not determined whether the associations between NPS and elevated brain amyloid differ by cognitive status (cognitively unimpaired (CU) vs. MCI), or have not conducted subanalyses among persons with amnestic MCI (aMCI). aMCI coupled with amyloid positivity is considered the typical prodromal stage of dementia due to AD^15^.

We sought to examine the frequency of NPS among community-dwelling non-demented participants (CU; MCI) by brain amyloid status (abnormal, A+ vs. normal, A−). We then investigated whether cognitive/amyloid status was associated with the odds of having NPS. We also conducted secondary analyses among CU and aMCI participants. Based on previous literature, we hypothesized that persons with MCI and elevated amyloid burden would have higher odds of having NPS as compared to MCI persons without elevated amyloid burden or CU participants.

## Materials and methods

### Design and sample

This cross-sectional study was derived from the ongoing, population-based Mayo Clinic Study of Aging (MCSA) in Olmsted County, MN^16^. Eligible participants were non-demented and ≥ 50 years of age, with a concurrent, valid NPS assessment and amyloid positron emission tomography (PET) neuroimaging. The institutional review boards of the Mayo Clinic and Olmsted Medical Center in Rochester, MN approved the MCSA protocols. All study participants provided written informed consent.

### Neurocognitive evaluation

Participants underwent a face-to-face evaluation including a neurological examination, a study coordinator visit, and neuropsychological testing. The reader is referred elsewhere for details on the face-to-face evaluation^16^. Briefly, the neurological evaluation comprised a neurological history review, administration of the Short Test of Mental Status^17^, and a neurological examination. The study coordinator visit included the Clinical Dementia Rating Scale (CDR)^18^. Neuropsychological testing was administered by a psychometrist in order to assess performance in four cognitive domains: memory (delayed recall trials from Auditory Verbal Learning Test^19^, Wechsler Memory Scale-Revised^20^, Logical Memory and Visual Reproduction subtests), language (Boston Naming Test^21^, category fluency^22^); visuospatial skills (Wechsler Adult Intelligence Scale-Revised^23^, Picture Completion and Block Design subtests); and attention/executive function (Trail-Making Test Part B^24^, Wechsler Adult Intelligent Scale-Revised^23^, Digit Symbol Substitution subtest). An expert consensus panel consisting of physicians, study coordinators, and neuropsychologists reviewed the results for each participant and determined whether a participant was CU or had MCI based on published criteria. Individuals were classified as CU based on normative data developed in this community^25–28^. For MCI, the revised Mayo Clinic criteria for MCI^29,30^ were used: (1) cognitive concern expressed by a physician, informant, participant, or nurse; (2) impairment in one or more cognitive domains (executive functions, memory, language, or visuospatial skills); (3) essentially normal functional activities; and (4) absence of dementia. Participants with MCI had a CDR score of 0 or 0.5; however, the final diagnosis of MCI was based on all available data.

### Assessment of NPS

We measured NPS using the Neuropsychiatric Inventory Questionnaire (NPI-Q)^31^. The NPI-Q is designed to assess 12 emotional behaviors (i.e., agitation, delusion, hallucination, depression, anxiety, euphoria, apathy, disinhibition, irritability, aberrant motor behavior, sleep, and eating/appetite) and was administered as a structured interview to an informant by a research nurse or study coordinator. In addition to the NPI-Q, participants completed the Beck Depression Inventory-II (BDI-II)^32^ and Beck Anxiety Inventory (BAI)^33^. Both inventories are validated and consist of 21 items that measure common symptoms of depression (such as feeling guilty or loss of interest) over the past 2 weeks and symptoms of anxiety (such as nervousness or fear of losing control) over the past 1 week. The severity of each symptom is rated on a Likert scale ranging from 0 to 3, with a total score ranging from 0 to 63 for BDI-II and BAI, respectively.

### Molecular imaging: PiB-PET acquisition

We performed amyloid PET imaging using the Pittsburgh Compound B tracer. The reader is referred elsewhere for details on PiB-PET imaging in the MCSA^34,35^. Briefly, PiB scans, consisting of four 5-min dynamic frames, were acquired from 40 to 60 min after intravenous injection with 292–728 MBq of ^11^C-PiB. Images were analyzed using an in-house, fully automated image processing pipeline in which image voxel values were extracted from automatically labeled regions of interest propagated from regions defined on each participant’s own magnetic resonance imaging (MRI). A global amyloid PET standardized uptake value ratio (SUVR) was formed from the prefrontal, orbitofrontal, parietal, temporal, anterior cingulate, and posterior cingulate/precuneus regions of interest and normalized to the cerebellar gray matter. Participants with an SUVR > 1.42 were classified as having an abnormal PiB-PET retention (elevated β-amyloid burden; A+) as we have previously validated by autopsy^36^.

### Statistical analysis

In this study, we created four groups based on cognitive/amyloid status: (1) CU with normal amyloid deposition (CU/A−; *n* = 997); (2) CU with elevated brain amyloid deposition (CU/A+; *n* = 446); (3) MCI with normal amyloid deposition (MCI/A−; *n* = 78); and (4) MCI with elevated brain amyloid deposition (MCI/A+; *n* = 106). In a first step, we compared baseline characteristics and frequency of NPS between these four groups, using Kruskal–Wallis and Fisher’s Exact tests (for continuous outcomes such as age; reported as median and interquartile range (IQR)) and Chi-square tests (for categorical outcomes such as male sex; reported as number and percentage, %). We then used multivariable logistic regression models to compute odds ratios (OR) and 95% confidence intervals (95% CIs) to compare the odds of having NPS between these four groups. In the regression analyses, we defined CU/A− persons as the reference group. NPS were the presumed dependent variables, and cognitive/amyloid status groups were the presumed independent variables. We ran the regression analyses on depression, anxiety, apathy, irritability, nighttime behavior, any NPS, any non-psychotic NPS (all measured through NPI-Q) and excluded other symptoms measured by NPI-Q (i.e., agitation, motor behavior, appetite/eating change, delusions, hallucinations, euphoria, disinhibition, and any psychotic NPS) due to low number of events. In order to examine the odds of clinically significant depression and anxiety, we also ran the analyses for BDI-II ≥ 13, BAI ≥ 8, and BAI ≥ 10. Finally, we conducted secondary analyses comparing CU (CU/A−; CU/A+) and amnestic MCI participants (aMCI/A−; aMCI/A+). All analyses were adjusted for traditional confounders, i.e., age, sex, education, as well as Apolipoprotein (APOE) ε4 genotype status. Analyses were done using the two-tailed alpha level of 0.05 and performed with SAS 9.4 (SAS Institute, Cary, NC).

## Results

### Demographics

The sample consisted of 1627 persons (54.3% males). The median (IQR) age was 72.7 (64.5, 79.3) years. Nine hundred and ninety-seven participants were CU/A−, 446 CU/A+, 78 MCI/A−, and 106 were MCI/A+. Four hundred and fifty-four participants (28.4%) were APOE ε4 carriers (Table 1). The MCI/A− group had a significantly higher percentage of males compared to the other groups. Participants who were MCI/A+ had the highest median age, and participants who were CU/A− had the lowest median age. For an overview of demographics for the CU and aMCI sample, please refer to Supplementary Table 1.

**Table 1:** Demographic characteristics of study participants

	CU/A− (*n* = 997)	CU/A+ (*n* = 446)	MCI/A− (*n* = 78)	MCI/A+ (*n* = 106)	Total (*n* = 1627)	*p*
Male sex, *N* (%)	539 (54.1)	232 (52.0)	59 (75.6)	54 (50.9)	884 (54.3)	**0.001** ^a^
Age, years	68.7 (61.3, 75.8)	77.2 (71.5, 82.1)	76.7 (69.3, 82.8)	81.3 (75.8, 86.0)	72.7 (64.5, 79.3)	**<** **0.001** ^b^
Age, *N* (%); years						**<** **0.001** ^a^
50–69	545 (54.7)	89 (20.0)	20 (25.6)	6 (5.7)	660 (40.6)	
70–95	452 (45.3)	357 (80.0)	58 (74.4)	100 (94.3)	967 (59.4)	
Education, years	15.0 (13.0, 16.0)	14.0 (12.0, 16.0)	12.0 (12.0, 16.0)	13.0 (12.0, 16.0)	14.0 (12.0, 16.0)	**<** **0.001** ^b^
> 12 years, *N* (%)	758 (76.0)	316 (70.9)	38 (48.7)	58 (54.7)	1170 (71.9)	**<** **0.001** ^a^
APOE ε4 carrier, *N* (%)	205 (21.0)^{19}^	183 (41.5)^{5}^	13 (17.1)^{2}^	53 (50.5)^{1}^	454 (28.4)^{27}^	**<** **0.001** ^a^

Data presented are median (interquartile range) unless otherwise noted. Significant *p* values appear bold

*CU* cognitively unimpaired, *MCI* mild cognitive impairment, *A−* normal PiB-PET, *A+* abnormal PiB-PET, *APOE ε4* Apolipoprotein ε4, ^*{N}*^ number of missing data

^a^Chi-Square test

^b^Kruskal–Wallis test

### Presence of NPS

The MCI/A+ group had the highest frequency of any NPS (59.4%), followed by MCI/A− (34.6%), CU/A+ (24.9%), and CU/A− (20.0%; Table 2). Psychotic symptoms were rare across groups. The MCI/A+ group had the highest frequency of persons with clinical depression (BDI-II ≥ 13) and clinical anxiety (BAI ≥ 10). Similarly, in the secondary analyses including aMCI and CU participants, the aMCI/A+ group had the highest frequency of any NPS (59.3%), followed by aMCI/A− (35.7%), CU/A+ (24.9%), and CU/A− (20%). Please refer to Supplementary Table 2 for display of data.

**Table 2:** Frequency of neuropsychiatric symptoms across cognitive/amyloid status groups

	CU/A− (*n* = 997)	CU/A+ (*n* = 446)	MCI/A− (*n* = 78)	MCI/A+ (*n* = 106)	Total (*n* = 1627)	*p*
Non-psychotic NPS						
Agitation	15 (1.5)	12 (2.7)	5 (6.4)	11 (10.4)	43 (2.6)	**<** **0.001** ^d^
Depression/dysphoria	100 (10.0)	51 (11.4)	10 (12.8)	31 (29.2)	192 (11.8)	**<** **0.001** ^e^
Anxiety	42 (4.2)	28 (6.3)	9 (11.5)	21 (19.8)	100 (6.1)	**<** **0.001** ^e^
Apathy	32 (3.2)	26 (5.8)	7 (9.0)	24 (22.6)	89 (5.5)	**<** **0.001** ^e^
Irritability	68 (6.8)	40 (9.0)	16 (20.5)	25 (23.6)	149 (9.2)	**<** **0.001** ^e^
Motor behavior	8 (0.8)	3 (0.7)	3 (3.8)	5 (4.7)	19 (1.2)	**0.002** ^d^
Nighttime behavior^a^	40 (4.5)	32 (7.9)	5 (7.0)	17 (19.5)	94 (6.4)	**<** **0.001** ^e^
Appetite/eating change	31 (3.1)	14 (3.1)	3 (3.8)	16 (15.1)	64 (3.9)	**<** **0.001** ^d^
Psychotic NPS						
Delusions	1 (0.1)	1 (0.2)	2 (2.6)	3 (2.8)	7 (0.4)	**<** **0.001** ^d^
Hallucinations	0 (0.0)	2 (0.4)	0 (0.0)	2 (1.9)	4 (0.2)	**0.008** ^d^
Euphoria	4 (0.4)	3 (0.7)	0 (0.0)	1 (0.9)	8 (0.5)	0.591^d^
Disinhibition	10 (1.0)	2 (0.4)	3 (3.8)	7 (6.6)	22 (1.4)	**<** **0.001** ^d^
Number of NPS (0–12)						**<** **0.001** ^f^
Mean (SD)	0.4 (0.9)	0.5 (1.0)	0.8 (1.5)	1.5 (1.9)	0.5 (1.1)	
Median (IQR)	0.0 (0.0, 0.0)	0.0 (0.0, 0.0)	0.0 (0.0, 1.0)	1.0 (0.0, 3.0)	0.0 (0.0, 0.0)	
Any NPS	199 (20.0)	111 (24.9)	27 (34.6)	63 (59.4)	400 (24.6)	**<** **0.001** ^e^
Any non-psychotic NPS	199 (20.0)	111 (24.9)	27 (34.6)	63 (59.4)	400 (24.6)	**<** **0.001** ^e^
Any psychotic NPS	12 (1.2)	8 (1.8)	3 (3.8)	10 (9.4)	33 (2.0)	**<** **0.001** ^d^
BDI-II total^b^						**<** **0.001** ^f^
Mean (SD)	4.1 (4.7)	4.6 (4.4)	4.9 (5.0)	7.5 (6.5)	4.5 (4.8)	
Median (IQR)	3.0 (1.0, 6.0)	4.0 (1.0, 7.0)	3.0 (1.0, 8.0)	7.0 (2.0, 9.0)	3.0 (1.0, 7.0)	
BDI-II ≥ 13^b^	54 (5.4)	28 (6.3)	8 (10.3)	17 (16.0)	107 (6.6)	**<** **0.001** ^e^
BAI total (0–63)^c^						**<** **0.001** ^f^
Mean (SD)	2.4 (3.7)	2.8 (4.3)	3.9 (5.2)	4.7 (5.5)	2.7 (4.1)	
Median (IQR)	1.0 (0.0, 3.0)	1.0 (0.0, 4.0)	2.0 (0.0, 6.0)	3.0 (1.0, 7.0)	1.0 (0.0, 4.0)	
BAI ≥ 8^c^	86 (8.6)	46 (10.3)	15 (19.2)	23 (21.7)	170 (10.5)	**<** **0.001** ^e^
BAI ≥ 10^c^	51 (5.1)	31 (7.0)	8 (10.3)	14 (13.2)	104 (6.4)	**0.004** ^e^

Data presented are *N* (%) unless otherwise noted. Significant *p* values appear bold

*CU* cognitively unimpaired, *MCI* mild cognitive impairment, *A−* normal PiB-PET, *A+* abnormal PiB-PET, *SD* standard deviation, *IQR* interquartile range, *BDI-II* Beck Depression Inventory II, *BAI* Beck Anxiety Inventory

^a^Information missing for 168 participants

^b^Information missing for 5 participants

^c^Information missing for 3 participants

^d^Fisher’s Exact test

^e^Chi-Square test

^f^Kruskal–Wallis test

### Association between NPS and cognitive/amyloid status

After adjusting for age, sex, education, and APOE ε4 carrier status, the odds of having any NPS as measured by NPI-Q (i.e. depression, anxiety, apathy, irritability, nighttime behavior, any NPS, and any non-psychotic NPS), depression (BDI-II ≥ 13), or anxiety (BAI ≥ 8, BAI ≥ 10) were significantly increased for the MCI/A+ group as compared to the reference group (CU/A−) for all variables (Table 3). MCI/A− participants had significantly increased odds of having anxiety, apathy, irritability, any NPS, any non-psychotic NPS, BAI ≥ 8, and BAI ≥ 10 as compared to the reference group. In the secondary analyses including CU and aMCI participants, there was a similar trend: aMCI/A+ participants, as compared to CU/A−, had significantly increased odds of having NPS (all NPI-Q variables), depression (BDI-II ≥ 13), or anxiety (BAI ≥ 8, BAI ≥ 10). For aMCI/A− participants, the odds were significantly increased for irritability, any NPS, any non-psychotic NPS, and BAI ≥ 8 (Supplementary Table 3).

**Table 3:** Odds of having neuropsychiatric symptoms by cognitive/amyloid status groups

Dependent variable	*N*	Independent variable	*N*	OR (95% CI)	*p*
Depression	97	CU/A−	978	1.00 (reference)	
	51	CU/A+	441	1.13 (0.76, 1.67)	0.55
	10	MCI/A−	76	1.33 (0.65, 2.72)	0.43
	31	MCI/A+	105	3.48 (2.05, 5.90)	**<** **0.001**
Anxiety	42	CU/A−	978	1.00 (reference)	
	27	CU/A+	441	1.45 (0.84, 2.50)	0.18
	9	MCI/A−	76	2.82 (1.27, 6.25)	**0.011**
	21	MCI/A+	105	5.29 (2.73, 10.23)	**<** **0.001**
Apathy	31	CU/A−	978	1.00 (reference)	
	26	CU/A+	441	1.62 (0.90, 2.91)	0.11
	7	MCI/A−	76	2.51 (1.03, 6.09)	**0.042**
	24	MCI/A+	105	7.06 (3.59, 13.88)	**<** **0.001**
Irritability	68	CU/A−	978	1.00 (reference)	
	39	CU/A+	441	1.32 (0.84, 2.08)	0.23
	16	MCI/A−	76	3.39 (1.80, 6.38)	**<** **0.001**
	25	MCI/A+	105	4.21 (2.34, 7.58)	**<** **0.001**
Nighttime behavior	39	CU/A−	879	1.00 (reference)	
	31	CU/A+	400	1.31 (0.77, 2.23)	0.32
	5	MCI/A−	69	1.13 (0.42, 3.06)	0.81
	17	MCI/A+	87	3.06 (1.52, 6.17)	**0.002**
Any NPS	192	CU/A−	978	1.00 (reference)	
	110	CU/A+	441	1.18 (0.88, 1.58)	0.27
	26	MCI/A−	76	1.77 (1.06, 2.97)	**0.029**
	63	MCI/A+	105	4.84 (3.06, 7.65)	**<** **0.001**
Any non-psychotic NPS	192	CU/A−	978	1.00 (reference)	
	110	CU/A+	441	1.18 (0.88, 1.58)	0.27
	26	MCI/A−	76	1.77 (1.06, 2.97)	**0.029**
	63	MCI/A+	105	4.84 (3.06, 7.65)	**<** **0.001**
BDI-II ≥ 13	53	CU/A−	977	1.00 (reference)	
	28	CU/A+	438	1.29 (0.77, 2.17)	0.33
	8	MCI/A−	76	1.70 (0.75, 3.87)	0.20
	17	MCI/A+	105	3.42 (1.75, 6.71)	**<** **0.001**
BAI ≥ 8	83	CU/A−	977	1.00 (reference)	
	45	CU/A+	440	1.22 (0.80, 1.86)	0.35
	15	MCI/A−	76	2.88 (1.52, 5.44)	**0.001**
	23	MCI/A+	105	2.97 (1.66, 5.30)	**<** **0.001**
BAI ≥ 10	50	CU/A−	977	1.00 (reference)	
	30	CU/A+	440	1.45 (0.86, 2.43)	0.16
	8	MCI/A−	76	2.31 (1.02, 5.26)	**0.045**
	14	MCI/A+	105	3.01 (1.47, 6.15)	**0.003**

Significant *p* values appear bold. Analyses adjusted for age, sex, education, and APOE ε4 genotype status

*CU* cognitively unimpaired, *MCI* mild cognitive impairment, *A−* normal PiB-PET, *A+* abnormal PiB-PET, *OR* odds ratio, *CI* confidence interval, *p* value comparing to reference group, *BDI-II* Beck Depression Inventory II, *BAI* Beck Anxiety Inventory

## Discussion

Here we report that community-dwelling older persons with coexistence of MCI and elevated brain amyloid deposition had significantly higher odds of having NPS as compared to CU persons without elevated brain amyloid deposition. For example, the odds of having apathy were 7.1 times higher in MCI/A+ than CU/A− persons. Similarly, the odds for MCI/A+ as compared to CU/A− persons of having anxiety were 5.3 times, of irritability 4.2 times, and of having depression 3.5 times higher. The same observations hold true for aMCI persons in our secondary analyses, i.e., the odds of having NPS for the aMCI/A+ group were significantly higher than for the reference group. Furthermore, on average, MCI/A− persons had the second highest odds of having NPS, and the odds were significantly increased for anxiety, apathy, irritability, any NPS, any non-psychotic NPS, BAI ≥ 8, and BAI ≥ 10 as compared to the reference group (CU/A−). These results changed slightly when we looked at aMCI/A− vs. CU/A− persons; only the increased odds for irritability, any NPS, any non-psychotic NPS, and BAI ≥ 8 remained significant. Furthermore, we additionally adjusted the analyses for global cognition (*z*-score) to see whether this would impact the findings. Indeed, we observed that the odds for MCI/A+ participants were still significantly increased for depression, anxiety, apathy, irritability, any NPS, any non-psychotic NPS, and BDI-II ≥ 13 but no longer remained significantly increased for nighttime behavior, BAI ≥ 8, and BAI ≥ 10 (data not shown). Similarly, the odds were still significantly increased for MCI/A− for irritability and BAI ≥ 8 but no longer for anxiety, apathy, any NPS, any non-psychotic NPS, and BAI ≥ 10 (data not shown).

Overall, our results were in line with our hypothesis that the combined presence of cognitive impairment and elevated brain amyloid deposition is associated with greater odds of having NPS as compared to MCI without amyloid deposition. This indicates that the underlying AD biology as measured by PET Aβ imaging contributes to an increased frequency of NPS over and above expected from MCI status alone, which implies that the underlying AD biology may drive both the cognitive symptoms and psychiatric symptoms. Of note, 78 of our participants were MCI/A-. The widely held interpretation is that the underlying neurobiological substrate of an amyloid negative MCI is most likely to be non-AD; whereas MCI with abnormal amyloid is interpreted as MCI due to AD^37^. In addition, even though the MCI/A+ group was about 10 years older than the CU/A− group, our findings are over and above what can be explained by age since we have rigorously adjusted for age.

Our findings should also be interpreted within the context of published literature. We and others have previously reported cross-sectional associations between NPS, particularly depression, anxiety, and apathy, with increased amyloid deposition in cognitively normal^6,8^ or cognitively impaired persons^10,11^, whereas few studies found no association^9^ or only in subgroup analyses^7^. To our knowledge, there is only a limited number of cross-sectional studies that examined the association between NPS and cortical amyloid in both cognitively normal and cognitively impaired persons. French researchers reported that, in a sample comprised of CU persons as well as MCI and AD patients, anxiety and irritability were associated with higher cortical amyloid deposition in various regions of interest; the number of significant correlations was reduced in subgroup analyses among MCI or AD only^12^. Similarly, researchers from UCLA observed that the relationship between depression and anxiety with amyloid deposition varied between cognitively normal and MCI persons, i.e., depression and anxiety in MCI were associated with amyloid deposition in different regions of interest than in cognitively normal persons^13^. Finally, a group of investigators from Pittsburgh found that PiB retention in three out of six depressed MCI patients was comparable to PiB retention in AD patients^14^. However, these studies differ from ours in various ways, i.e., our study had a larger, population-based sample, and we wanted to know whether combined presence of cognitive impairment and abnormal amyloid deposition was associated with the odds of having NPS. Our study is also in line with a recently published longitudinal study from Harvard Medical School reporting that higher amyloid beta burden was associated with increasing anxious-depressive symptoms over time in cognitively normal older individuals^38^. Our current study may also be relevant to the ISTAART-AA criteria for mild behavioral impairment (MBI) and the recently published MBI checklist (MBI-C)^39^. The construct of MBI refers to a change in behavior or personality in persons without dementia aged ≥ 50 years that may be the case in a majority of our study population.

The strengths of our study are the large-scale, population-based sample and the rigorous acquisition of NPS using three validated assessments that are widely used in aging research. The limitations pertain to the relatively small number of participants reporting NPS. However, this was expected given that participants are community-dwelling individuals aged ≥ 50 years and similar frequencies of NPS in community-based samples have been reported by our group and others in the past^40,41^. Owing to the low number, we were not able to conduct regression analyses stratified by APOE ε4 status, but we adjusted our analyses for this potential confounder. Furthermore, in order to avoid an assumption violation of the logistic regression, we did not run the analyses on all NPS that are listed in Table 2 and Supplementary Table 2, i.e., we excluded agitation, motor behavior, appetite change, delusions, hallucinations, euphoria, disinhibition, and any psychotic NPS due to low number of events. Another limitation is that we did not take into account the severity of NPS as assessed by the NPI-Q and instead only examined the presence vs. absence of symptoms. However, we included clinical depression and anxiety as measured by BDI-II (score ≥ 13) and BAI (score ≥ 8; score ≥ 10) in our analyses, even though the scales do not take into consideration whether the symptoms are acute or chronic. For example, a person who has recurrent depression and is successfully treated with antidepressant medication may be symptom-free within 2 weeks of completing the BDI-II questionnaire. We also did not include or adjust the analyses for other neuroimaging biomarkers such as structural MRI of hippocampal volumes and frontal/global cortical atrophy, and we did not investigate various regions of interest but rather a global amyloid PET SUVR. In addition, we did not adjust our analyses for multiple comparisons. However, when considering a Bonferroni correction for Table 3, the alpha significance level would be 0.00167 (i.e., 0.05/30 since we have a total of 10 models with 3 comparisons per model). Thus 10 out of the 17 significant *p* values still remain significant and none of our major conclusions are affected by the correction. Finally, given the cross-sectional nature of our study, the direction of causality between NPS with amyloid and cognitive status cannot be determined. The current study did not investigate underlying mechanisms that may explain the association between cognitive/amyloid status with NPS. We and others have previously proposed potential mechanisms linking NPS with cognitive outcomes and neuroimaging biomarkers, such as etiologic pathway, shared risk factor or confounding, reverse causality, and/or interaction^42,43^.

Our cross-sectional data show that community-dwelling persons with combined presence of MCI and amyloid positivity as measured by PiB-PET are at significantly increased risk of having NPS. These findings add valuable information on the association between NPS and cognitive/amyloid status. Furthermore, they underline the importance of a thorough assessment as well as potential treatment of NPS in the clinical practice, particularly among persons with confirmed neuroimaging biomarker abnormality who are at risk for AD. The findings need to be confirmed by a prospective cohort study.

## Supplementary information

Supplementary Table 1.

Supplementary Table 2.

Supplementary Table 3.
