# Supplementary material for: Cortical β-amyloid burden, neuropsychiatric symptoms, and cognitive status: the Mayo Clinic Study of Aging
Source: Transl Psychiatry. 2019 Mar 28;9:123. doi: 10.1038/s41398-019-0456-z (PMC6438979; doi:10.1038/s41398-019-0456-z)
Supplement: Supplementary file 1 — Supplementary Table 1. [file 41398_2019_456_MOESM1_ESM.docx]

**Supplementary Table 1: Demographic characteristics of study participants**

|  | **CU/A-**  **N = 997** | **CU/A+ N = 446** | **aMCI/A- N = 56** | **aMCI/A+ N = 86** | **Total N = 1585** | **p** |
| --- | --- | --- | --- | --- | --- | --- |
| Male sex, N (%) | 539 (54.1) | 232 (52.0) | 45 (80.4) | 48 (55.8) | 864 (54.5) | **0.001^1^** |
| Age, years | 68.7  (61.3, 75.8) | 77.2  (71.5, 82.1) | 77.0  (70.7, 83.0) | 81.7  (76.1, 85.9) | 72.6  (64.3, 79.1) | **<0.001^2^** |
| Age, N (%) |  |  |  |  |  | **<0.001^1^** |
| 50-69 | 545 (54.7) | 89 (20.0) | 13 (23.2) | 3 (3.5) | 650 (41.0) |  |
| 70-95 | 452 (45.3) | 357 (80.0) | 43 (76.8) | 83 (96.5) | 935 (59.0) |  |
| Education, years | 15.0  (13.0, 16.0) | 14.0  (12.0, 16.0) | 13.0  (12.0, 16.0) | 13.0  (12.0, 16.0) | 14.0  (12.0, 16.0) | **<0.001^2^** |
| > 12 years, N (%) | 758 (76.0) | 316 (70.9) | 29 (51.8) | 47 (54.7) | 1150 (72.6) | **<0.001^1^** |
| APOE ε4 carrier, N (%) | 205 (21.0)^{19}^ | 183 (41.5)^{5}^ | 7 (13.0)^{2}^ | 47 (55.3)^{1}^ | 442 (28.4) ^{27}^ | **<0.001^1^** |

Data presented are median (interquartile range) unless otherwise noted. CU = cognitively unimpaired; aMCI = amnestic mild cognitive impairment; A- = normal PiB-PET; A+ = abnormal PiB-PET; APOE ε4 = Apolipoprotein ε4. ^1^ = Chi-Square test; ^2^ = Kruskal Wallis test; ^{N}^ = number of missing data. Significant p-values appear bold.
