# Supplementary material for: Cortical β-amyloid burden, neuropsychiatric symptoms, and cognitive status: the Mayo Clinic Study of Aging
Source: Transl Psychiatry. 2019 Mar 28;9:123. doi: 10.1038/s41398-019-0456-z (PMC6438979; doi:10.1038/s41398-019-0456-z)
Supplement: Supplementary file 2 — Supplementary Table 2. [file 41398_2019_456_MOESM2_ESM.docx]

**Supplementary Table 2: Frequency of neuropsychiatric symptoms across cognitive/ amyloid status groups**

|  | **CU/A-**  **N = 997** | **CU/A+ N = 446** | **aMCI/A- N = 56** | **aMCI/A+ N = 86** | **Total N = 1585** | **p** |
| --- | --- | --- | --- | --- | --- | --- |
| *Non-psychotic NPS* |  |  |  |  |  |  |
| Agitation | 15 (1.5) | 12 (2.7) | 2 (3.6) | 8 (9.3) | 37 (2.3) | **0.001^3^** |
| Depression/Dysphoria | 100 (10.0) | 51 (11.4) | 5 (8.9) | 25 (29.1) | 181 (11.4) | **<0.001^1^** |
| Anxiety | 42 (4.2) | 28 (6.3) | 5 (8.9) | 15 (17.4) | 90 (5.7) | **<0.001^3^** |
| Apathy | 32 (3.2) | 26 (5.8) | 5 (8.9) | 19 (22.1) | 82 (5.2) | **<0.001^3^** |
| Irritability | 68 (6.8) | 40 (9.0) | 11 (19.6) | 20 (23.3) | 139 (8.8) | **<0.001^1^** |
| Motor behavior | 8 (0.8) | 3 (0.7) | 3 (5.4) | 5 (5.8) | 19 (1.2) | **<0.001^3^** |
| Nighttime behavior* | 40 (4.5) | 32 (7.9) | 2 (4.0) | 14 (20.0) | 88 (6.2) | **<0.001^3^** |
| Appetite/ eating change | 31 (3.1) | 14 (3.1) | 2 (3.6) | 15 (17.4) | 62 (3.9) | **<0.001^3^** |
| *Psychotic NPS* |  |  |  |  |  |  |
| Delusions | 1 (0.1) | 1 (0.2) | 2 (3.6) | 2 (2.3) | 6 (0.4) | **0.001^3^** |
| Hallucinations | 0 (0.0) | 2 (0.4) | 0 (0.0) | 1 (1.2) | 3 (0.2) | **0.044^3^** |
| Euphoria | 4 (0.4) | 3 (0.7) | 0 (0.0) | 0 (0.0) | 7 (0.4) | 0.836^3^ |
| Disinhibition | 10 (1.0) | 2 (0.4) | 2 (3.6) | 4 (4.7) | 18 (1.1) | **0.006^3^** |
| Number of NPS (0-12) |  |  |  |  |  | **<0.001^2^** |
| Mean (SD) | 0.4 (0.9) | 0.5 (1.0) | 0.7 (1.3) | 1.5 (1.8) | 0.5 (1.0) |  |
| Median (IQR) | 0.0 (0.0, 0.0) | 0.0 (0.0, 0.0) | 0.0 (0.0, 1.0) | 1.0 (0.0, 3.0) | 0.0 (0.0, 0.0) |  |
| Any NPS | 199 (20.0) | 111 (24.9) | 20 (35.7) | 51 (59.3) | 381 (24.0) | **<0.001^1^** |
| Any non-psychotic NPS | 199 (20.0) | 111 (24.9) | 20 (35.7) | 51 (59.3) | 381 (24.0) | **<0.001^1^** |
| Any psychotic NPS | 12 (1.2) | 8 (1.8) | 2 (3.6) | 7 (8.1) | 29 (1.8) | **0.001^3^** |
| BDI-II total^ |  |  |  |  |  | **<0.001^2^** |
| Mean (SD) | 4.1 (4.7) | 4.6 (4.4) | 4.3 (4.7) | 7.2 (6.5) | 4.4 (4.8) |  |
| Median (IQR) | 3.0 (1.0, 6.0) | 4.0 (1.0, 7.0) | 3.0 (1.0, 5.5) | 6.0 (3.0, 9.0) | 3.0 (1.0, 6.0) |  |
| BDI-II ≥ 13^ | 54 (5.4) | 28 (6.3) | 4 (7.1) | 12 (14.0) | 98 (6.2) | **0.019^1^** |
| BAI total (0-63)^†^ |  |  |  |  |  | **<0.001^2^** |
| Mean (SD) | 2.4 (3.7) | 2.8 (4.3) | 3.3 (4.0) | 4.4 (5.3) | 2.6 (4.0) |  |
| Median (IQR) | 1.0 (0.0, 3.0) | 1.0 (0.0, 4.0) | 2.0 (0.0, 5.0) | 3.0 (1.0, 6.0) | 1.0 (0.0, 4.0) |  |
| BAI ≥ 8^†^ | 86 (8.6) | 46 (10.3) | 10 (17.9) | 17 (19.8) | 159 (10.1) | **0.002^1^** |
| BAI ≥ 10^†^ | 51 (5.1) | 31 (7.0) | 4 (7.1) | 9 (10.5) | 95 (6.0) | 0.154^1^ |

Data presented are N (%) unless otherwise noted. CU = cognitively unimpaired; aMCI = amnestic mild cognitive impairment; A- = normal PiB-PET; A+ = abnormal PiB-PET; SD = standard deviation; IQR = interquartile range; BDI-II = Beck Depression Inventory II, BAI = Beck Anxiety Inventory. ^1^ = Chi-Square test; ^2^ = Kruskal Wallis test; ^3^ = Fisher’s Exact test. * Information missing for 164 participants; ^ Information missing for 5 participants; **^†^** Information missing for 3 participants. Significant p-values appear bold.
