# Supplementary material for: Cortical β-amyloid burden, neuropsychiatric symptoms, and cognitive status: the Mayo Clinic Study of Aging
Source: Transl Psychiatry. 2019 Mar 28;9:123. doi: 10.1038/s41398-019-0456-z (PMC6438979; doi:10.1038/s41398-019-0456-z)
Supplement: Supplementary file 3 — Supplementary Table 3. [file 41398_2019_456_MOESM3_ESM.docx]

**Supplementary Table 3: Odds of having neuropsychiatric symptoms by cognitive/ amyloid status group**

| **Dependent variable** | **N** | **Independent variable** | **N** | **OR (95% CI)** | **p** |
| --- | --- | --- | --- | --- | --- |
| Depression | 97 | CU/A- | 978 | 1.00 (reference) |  |
|  | 51 | CU/A+ | 441 | 1.11 (0.75, 1.66) | 0.59 |
|  | 5 | aMCI/A- | 54 | 0.91 (0.35, 2.39) | 0.85 |
|  | 25 | aMCI/A+ | 85 | 3.40 (1.91, 6.04) | **<0.001** |
| Anxiety | 42 | CU/A- | 978 | 1.00 (reference) |  |
|  | 27 | CU/A+ | 441 | 1.49 (0.86, 2.58) | 0.16 |
|  | 5 | aMCI/A- | 54 | 2.30 (0.84, 6.30) | 0.10 |
|  | 15 | aMCI/A+ | 85 | 4.79 (2.29, 10.01) | **<0.001** |
| Apathy | 31 | CU/A- | 978 | 1.00 (reference) |  |
|  | 26 | CU/A+ | 441 | 1.58 (0.87, 2.84) | 0.13 |
|  | 5 | aMCI/A- | 54 | 2.54 (0.92, 7.02) | 0.073 |
|  | 19 | aMCI/A+ | 85 | 6.32 (3.06, 13.06) | **<0.001** |
| Irritability | 68 | CU/A- | 978 | 1.00 (reference) |  |
|  | 39 | CU/A+ | 441 | 1.30 (0.82, 2.05) | 0.26 |
|  | 11 | aMCI/A- | 54 | 3.22 (1.54, 6.72) | **0.002** |
|  | 20 | aMCI/A+ | 85 | 3.96 (2.09, 7.48) | **<0.001** |
| Nighttime behavior | 39 | CU/A- | 879 | 1.00 (reference) |  |
|  | 31 | CU/A+ | 400 | 1.28 (0.75, 2.19) | 0.37 |
|  | 2 | aMCI/A- | 48 | 0.63 (0.14, 2.76) | 0.54 |
|  | 14 | aMCI/A+ | 70 | 3.01 (1.42, 6.42) | **0.004** |
| Any NPS | 192 | CU/A- | 978 | 1.00 (reference) |  |
|  | 110 | CU/A+ | 441 | 1.17 (0.87, 1.58) | 0.29 |
|  | 19 | aMCI/A- | 54 | 1.87 (1.03, 3.40) | **0.039** |
|  | 51 | aMCI/A+ | 85 | 4.76 (2.89, 7.85) | **<0.001** |
| Any non-psychotic NPS | 192 | CU/A- | 978 | 1.00 (reference) |  |
|  | 110 | CU/A+ | 441 | 1.17 (0.87, 1.58) | 0.29 |
|  | 19 | aMCI/A- | 54 | 1.87 (1.03, 3.40) | **0.039** |
|  | 51 | aMCI/A+ | 85 | 4.76 (2.89, 7.85) | **<0.001** |
| BDI-II ≥ 13 | 53 | CU/A- | 977 | 1.00 (reference) |  |
|  | 28 | CU/A+ | 438 | 1.31 (0.77, 2.20) | 0.32 |
|  | 4 | aMCI/A- | 54 | 1.21 (0.41, 3.60) | 0.73 |
|  | 12 | aMCI/A+ | 85 | 3.01 (1.41, 6.42) | **0.004** |
| BAI ≥ 8 | 83 | CU/A- | 977 | 1.00 (reference) |  |
|  | 45 | CU/A+ | 440 | 1.24 (0.81, 1.90) | 0.32 |
|  | 10 | aMCI/A- | 54 | 2.81 (1.33, 5.97) | **0.007** |
|  | 17 | aMCI/A+ | 85 | 2.75 (1.44, 5.26) | **0.002** |
| BAI ≥ 10 | 50 | CU/A- | 977 | 1.00 (reference) |  |
|  | 30 | CU/A+ | 440 | 1.43 (0.85, 2.41) | 0.18 |
|  | 4 | aMCI/A- | 54 | 1.68 (0.56, 4.99) | 0.35 |
|  | 9 | aMCI/A+ | 85 | 2.32 (1.01, 5.33) | **0.048** |

CU = cognitively unimpaired; aMCI = amnestic mild cognitive impairment; A- = normal PiB-PET; A+ = abnormal PiB-PET; OR = odds ratio; CI = con­fidence interval; p = p-value for comparing to reference group; BDI-II = Beck Depression Inventory II, BAI = Beck Anxiety Inventory. Significant p-values appear bold. Analyses adjusted for age, sex, education, and APOE ε4 genotype status.
